# Is It Time to Address Burnout in the Military? Initial Psychometric Validation of the Maslach Burnout Inventory Among Tunisian Military Personnel (A-MBI-MP)

**DOI:** 10.3390/bs15030385

**Published:** 2025-03-19

**Authors:** Ghada Boussayala, Amayra Tannoubi, John Elvis Hagan, Mustapha Amoadu, Medina Srem-Sai, Tore Bonsaksen, Hamdi Henchiri, Mohamed Karim Chtioui, Lotfi Bouguerra, Fairouz Azaiez

**Affiliations:** 1 Higher Institute of Sport and Physical Education of Sfax, University of Sfax, Sfax 3000, Tunisia; ghada.boussayala@issepsf.u-sfax.tn (G.B.); hamdi.henchiri@issepsf.u-sfax.tn (H.H.); 2 Higher Institute of Sport and Physical Education of Gafsa, University of Gafsa, Gafsa 2100, Tunisia; amayra.tannoubi@issepgf.ugaf.tn (A.T.); fairouz.azaiez@issepgf.ugaf.tn (F.A.); 3 Sports Performance Optimization Research Laboratory (LR09SEP01), National Center for Sports Medicine and Science (CNMSS), Tunis 1003, Tunisia; 4 Neurocognition and Action-Biomechanics-Research Group, Faculty of Psychology and Sports Science, Bielefeld University, Postfach 10 01 31, 33501 Bielefeld, Germany; 5 Department of Health, Physical Education and Recreation, University of Cape Coast, Cape Coast PMB TF0494, Ghana; 6 Biomedical and Clinical Research Centre, University of Cape Coast, Cape Coast PMB CC3321, Ghana; mustapha.amoadu@ucc.edu.gh; 7 Department of Health, Physical Education, Recreation and Sports, University of Education, Winneba P.O. Box 25, Ghana; mssai@uew.edu.gh; 8 Department of Health and Nursing Science, Faculty of Social and Health Sciences, Inland Norway University of Applied Sciences, 2406 Elverum, Norway; tore.bonsaksen@inn.no; 9 Department of Health, Faculty of Health Studies, VID Specialized University, 4024 Stavanger, Norway; 10 General Direction of Military Health, Tunis 1000, Tunisia; medkarim_chtioui@hotmail.fr; 11 Department of Physiology, Faculty of Sciences of Bizerte, University of Carthage, Bizerte 7053, Tunisia; lotfibouguerra@yahoo.fr

**Keywords:** Arabic context, burnout, depersonalization, emotional exhaustion, Maslach Burnout Inventory, military, personal accomplishment

## Abstract

The military’s high-pressure environment can lead to burnout syndrome, characterized by emotional fatigue, depersonalization, and decreased personal accomplishment. Validating a culturally appropriate tool for assessing burnout among military personnel is crucial for early detection and intervention. This study assessed the psychometric properties of the Arabic version of the Maslach Burnout Inventory (MBI) and its validity among Tunisian military personnel. A validation study was conducted among 520 Tunisian military personnel (mean age = 36 ± 9.3 years; male (n = 486) and female (n = 34)), including commandos, pilots, and divers. The Arabic version of the Maslach Burnout Inventory MBI-HSS was administered, including participants’ sociodemographic characteristics. The exploratory (EFA) and confirmatory (CFA) factor analyses were performed to identify the factor structure, with assessments of the internal consistency of the model. The factor analysis confirmed the three-factor model of burnout: emotional exhaustion, depersonalization, and personal accomplishment consistent with the original MBI. The A-MBI-MP demonstrated strong internal consistency, with Cronbach’s alpha values exceeding 0.8 for all subscales. The test–retest reliability was also excellent, confirming the stability of the instrument. The CFA validated the three-factor structure of the A-MBI-MP. The fit indices demonstrated an acceptable model fit: Comparative Fit Index (CFI) = 0.949, Tucker–Lewis Index (TLI) = 0.943, and Root Mean Square Error of Approximation (RMSEA) = 0.0742 (95% CI: 0.068–0.0797). These results validate the reliability of the A-MBI-MP in assessing burnout in the military context. The Arabic version of the Maslach Burnout Inventory (A-MBI-MP) is a valid and reliable tool for assessing burnout among Tunisian military personnel. The validated instrument can be used to offer early treatments and to promote the mental health of military personnel in high-stress situations.

## 1. Introduction

Burnout, which is defined as emotional exhaustion, depersonalization, and an overall decrease in personal accomplishment (Rumschlag, 2017), has become a major issue that affects people in a variety of professional fields (Maslach & Leiter, 2016). Numerous causes that might lead to burnout are related to the workplace, with well-known precursors such as excessive workload, high levels of workplace stress, a lack of effort and rewards, and strained collegial relationships (Detaille et al., 2020). According to the World Health Organization, burnout is a serious professional phenomenon that may negatively affect employees’ well-being at work (World Health Organization, 2019). Schaufeli et al. (2020) assert that persistent workplace stress, which has not been adequately managed, is considered to be the primary cause of burnout syndrome (Schaufeli et al., 2020). It has three dimensions: feelings of energy depletion, increased mental distance from one’s job, and reduced professional efficacy (Maslach & Jackson, 1981).

Burnout as a concept has been part of the popular vocabulary for the better part of a century and perhaps even longer (Han, 2015). The concept of human stress response to difficult life events (stressors) was developed in the 1950s (Mason, 1975). Earlier, the concept of burnout was most commonly used in engineering to describe the result when repetitive stress or excessive load on a piece of equipment ruins its ability to function, as when a motor, light bulb, or rocket booster burns out (Etzion, 1988).

Due to the multidimensional and complex character of burnout, it is usually evaluated by numerous approved instruments, each aimed at measuring different aspects of the syndrome (Schaufeli et al., 2017). There are various tools that have been designed to evaluate this phenomenon, such as the Copenhagen Burnout Inventory (CBI) (Kristensen et al., 2005), Oldenburg Burnout Inventory (OBI) (Demerouti et al., 2003), Questionnaire for the Evaluation of Burnout Syndrome (CESQT) (Gil-Monte, 2011), and Burnout Assessment Tool (BAT) (Schaufeli et al., 2020). However, the MBI remains the most prevalent and validated assessment to measure burnout (Maslach et al., 1997). Considering the MBI’s robust psychometric properties, including reliability, validity, and factor structure, it is a scientifically valid selection for this study due to its extensive usage (Schaufeli & Enzmann, 2020). The internal consistency is strong, with Cronbach’s alpha values exceeding 0.7 for the three subscales: emotional exhaustion, depersonalization, and personal accomplishment. The test–retest reliability indicates that the instrument demonstrates temporal stability (Leiter & Schaufeli, 1996). Furthermore, a meta-analysis by Wheeler et al. showed that the emotional exhaustion subscale demonstrates robust temporal reliability, even though the depersonalization and personal accomplishment subscales display moderate stability (Wheeler et al., 2011). The three-factor model of the Maslach Burnout Inventory has been confirmed across many populations and jobs. The inventory‘s ability to differentiate burnout from other psychological variables, including depression and stress, further indicates its validity (Maslach et al., 2001).

To explain the mechanisms and factors that contribute to burnout, different models have been suggested. In the one-factor model, burnout has been defined as “The inability to find existential meaning in one’s work”, so many people saw work as an existential quest. If this quest fails, burnout sets in (Pines & Keinan, 2005). According to Pines and colleagues, burnout is caused by a deeper factor that authorizes the person to seek his job. The more an individual enters with a strong involvement in a profession, the greater the probability that he will be a victim of burnout if the work conditions are unfavorable (Delobbe et al., 2005). The two-factor model defined burnout as a persistent imbalance of demands over resources (Bakker et al., 2007). Generally, there are two types of variables in any kind of employment: job demands and job resources. Jones and Fletcher (Jones & Fletcher, 1993) defined demands as “the things that have to be done. It is evident that in every job a task needs to be finished”. Job resources refer to physical, psychological, social, or organizational aspects of a job that, firstly, reduce job demands and the psychological costs associated with them, secondly, work ideally to achieve objectives, and finally, stimulate personal development and progress (Hobfoll, 2002). In the three-factor model developed by Christina Maslach and her colleagues, the Maslach Burnout Inventory (MBI) is commonly acknowledged as the primary tool for measuring and estimating burnout. The three primary aspects of burnout are described as follows: emotional exhaustion, considered the fundamental element of burnout, as it reflects the profound fatigue that individuals feel, and it is a dominant predictor of burnout-related effects like work dissatisfaction and absenteeism (Maslach et al., 2001); depersonalization, which leads to diminished empathy and participation, characterized by detachment and potential frustration (Maslach et al., 1997); and personal accomplishment, which reflects feelings of incompetence, failure, and a lack of achievement in one’s work (Schaufeli & Enzmann, 2020).

While great research has been conducted about burnout in different contexts such as health care providers (Dubale et al., 2019; Hassankhani et al., 2024; Henchiri et al., 2025; Méndez et al., 2024; Pradas-Hernández et al., 2018), teachers (García-Carmona et al., 2019; Wang et al., 2025), and students (IsHak et al., 2013; López-Gómez et al., 2025), it is crucial to understand its manifestations and impact on military operational personnel. In the dynamic environment of military operations, the mental health of the personnel is crucial to missions’ success. Military personnel face difficulties because of the nature of work characterized by stressful situations, prolonged deployments, and a constant need for optimal performance, which presents difficulties for military personnel’s mental health (Wadsworth & Southwell, 2011). For this, understanding burnout in the military is essential, not only for the well-being of individual service members but also for the personal health of military operations (Ivie & Garland, 2011). Among all the psychological concepts that influence military personnel, burnout has emerged as an important subject of concern (Adler et al., 2017). This syndrome can have significant consequences on military personnel’s mental health that affect the success of their mission and the cohesion of their team.

Although the issue of burnout in military populations is becoming more and more recognized worldwide (Adler & Castro, 2013), there is a lack of empirical research addressing burnout within Tunisian military personnel. Mainly in the first decade after the 2011 Arab uprisings, Tunisia became a target of terrorist attacks executed by local and regional elements. What it takes to create a counter-terrorism strategy is characterized by an ensemble of measures employed by military forces to limit and terminate the operations of terrorist organizations or individuals (Santini & Cimini, 2019).

These military personnel are often confronted with distinct psychic, social, and environmental difficulties that may increase burnout (Lutterbeck, 2013). Consequently, validating the MBI for this specific cohort ensures its capacity to correctly represent the unique pressures and experiences of Tunisian military personnel. A tool lacking cultural and contextual validation may inadequately detect critical burnout symptoms, resulting in an incorrect diagnosis or an underestimation of the problem (Schou et al., 2012). Cultural norms that value collectivism, military hierarchies, and regional stresses may all play a role in causing burnout in Tunisia in ways that are distinct from those in Western countries (Salmoni & Holmes-Eber, 2008). Therefore, cultural validation is required in order to prevent incorrect diagnoses, which may result in poor interventions (Huang & Wong, 2023).

Thus, this study validated the Maslach Burnout Inventory in the context of military personnel in terms of factor structure, construct validity, and reliability to enhance the precision of the diagnosis. The study outcomes will also enable the creation of targeted interventions that align with the psychological and professional conditions of Tunisian military personnel.

## 2. Materials and Methods

### 2.1. Study Design

A validation study design was conducted to assess the reliability and validity of the Maslach Burnout Inventory (MBI) among military operational personnel (pilots, commandos, and divers) from February to December 2023. This design was chosen for its thorough methodology, addressing each aspect of the scientific process, including diligent planning, acceptable sample size determination, data collection, and the assessment of reliability and validity by several statistical techniques (Arafat, 2016).

### 2.2. Participants

The study sample comprised 214 commandos, 203 pilots, and 103 divers, all of whom were actively serving from February to December 2023. Participants were chosen through a stratified sample method (Hu et al., 2016) to guarantee participation from each subgroup of operational military personnel pertinent to the study. This resulted in a total of 520 participants.

#### Eligibility Criteria

Only participants who had served continuously for at least one month prior to the data collection were deemed eligible to guarantee a sample representative of active-duty troops experiencing standard operational stress. This approach was to ensure that the results of the study came from participants who were sufficiently exposed to military operations, work-related stressors, and routines.

Individuals who had been on vacation for more than one month before the research started were excluded.

### 2.3. Procedures

A member of the research team addressed qualified military personnel who were actively engaged in operations, explained the goal of the study, and requested that they complete an anonymous, self-reported questionnaire. They were also made aware that participation was entirely voluntary, both verbally and through written consent. Participants were free to decline or leave the study at any time with any consequences. The questionnaire was distributed to 520 military personnel at their places of employment following the receipt of signed informed consent.

### 2.4. Instrument

The original version of the MBI was developed by Cristina Maslach and Susan Jakson (Maslach et al., 1997). It contains 22 items on a seven (7)-point Likert scale. The participants have to indicate their emotions, ranging from (0) “never having this feeling” to (6) “having this feeling daily”. It measured burnout with three dimensions: emotional exhaustion (EE), which included nine items; depersonalization (DP), which was assessed through five items; and personal accomplishment (PA), which was measured through eight items. As a result, when participants have a high score of emotional exhaustion and depersonalization, it indicates a higher degree of burnout; however, a high score of personal accomplishment indicates a lower degree of burnout. Data collected through the survey contained the Arabic version of the MBI (A-MBI) and the basic sociodemographic and professional attributes of participants. Maslach and Jackson (Maslach & Jackson, 1981), in their original version of the MBI-SS, reported acceptable reliability coefficients, estimated by Cronbach’s alpha values for the instrument’s three subscales, 0.89, 0.74, and 0.59, respectively, for emotional exhaustion, personal accomplishment, and depersonalization.

#### Cross-Cultural Adaptation and Translation

To adapt the English version of the MBI-HSS (Lheureux et al., 2017) to the Arabic language, we obtained permission from Mind Garde, Inc. to translate the original MBI questionnaire. We sought help from two independent bilingual translators whose native language is Arabic, who translated the original English version of the MBI-HSS (Córdoba et al., 2011). They were directed to refrain from employing literal translation and rather use easy and comprehensible language for the military personnel. The first Arabic version was later translated back, and any differences in the phrasing compared to the original were examined by a group of specialists, which included military personnel and English and Arabic language instructors. The committee members engaged in deliberations and resolved conflicts to ensure semantic, idiomatic, and conceptual equivalence between the original instrument and the translated version. Content correspondence was the major criterion used to reach a consensus on the final wording of the item. A pre-test was conducted exclusively to discover any ambiguity in comprehending the questions. Before conducting the primary study, an initial version of the final scale was created and evaluated independently by a small group of 15 military personnel. The objective was to guarantee clarity in the scale items and facilitate responding. The pre-test group indicated no difficulties, and consequently, no changes were made to the scale. Cultural nuances were taken into consideration when translating and modifying the scale for our research population. This was performed by making sure that military terminology was contextualized accurately, modifying language particular to a certain position, and coordinating communication methods with hierarchical norms. Additionally, relevance is ensured while preventing misunderstandings by being mindful of historical background, ethical viewpoints, and regional conflicts. Furthermore, field tests with native military personnel guaranteed precision, efficacy, and conformity to the customs and values of the target culture.

### 2.5. Research Ethics Statement

Ethical approval for this study was obtained from the scientific committee of the local personnel protection committee at the general directorate of military health (001/2025/CLPP) dated 1 January 2025, the local ethics committee of the High Institute of Sport and Physical Education of Sfax, University of Sfax, Tunisia, and the local ethics committee of the High Institute of Sport and Physical Education of El Kef, University of Jendouba, Jendouba, Tunisia (Sp-0029/2024) dated 25 February 2024. The research was also deemed to comply with the latest norms of the Declaration of Helsinki 2024 (World Medical Association, 2024).

### 2.6. Data Analysis

The psychometric characteristics of the Arabic-Maslach Burnout Inventory for Military Personnel (A-MBI-MP) were evaluated by a series of analyses using the Statistical Package for Social Sciences (SPSS) software version 23 and AMOS version 22. Once the normality of the data distribution was verified, the Cronbach’s alpha coefficient was determined to assess the scale’s internal consistency. To validate the data, an exploratory factor analysis (EFA) and confirmatory analysis (CFA) were conducted to provide evidence for the validity of the instrument. The Kaiser–Meyer–Olkin (KMO) test and Bartlett’s test of sphericity were used to evaluate the sampling adequacy. The criterion used was to include components with eigenvalues greater than 1. Additionally, an orthogonal rotation (varimax) was applied. The factor structure of the measure (A-MBI-MP) was first examined using exploratory factor analysis (EFA). This was followed by a confirmatory factor analysis (CFA) where the factor loadings were assessed to evaluate the strength of the relationships between observed variables and latent factors, with loadings of 0.60 or higher considered acceptable indicators of good construct validity (Hair et al., 2014). Model fit indices, including the Comparative Fit Index (CFI) and Tucker–Lewis Index (TLI), were also evaluated; values above 0.90 for both indices indicate an adequate fit, and values above 0.95 indicate an excellent (Taasoobshirazi & Wang, 2016). The internal consistency of the A-MBI-MP subscales was assessed using Cronbach’s alpha, with values above 0.70 considered satisfactory (Tavakol & Dennick, 2011). Thirty (30) military personnel completed the MBI twice, with a 14-day gap between each test, to assess the test–retest reliability of the Arabic version of the questionnaire. For the analysis, we used the intra-class correlation coefficient (ICC; average measure). ICC values were interpreted as follows: 0.40–0.59 as fair, 0.60–0.74 as good, and 0.75–1.0 as excellent. All statistical tests used were two-tailed with a significance threshold of 0.05.

## 3. Results

### 3.1. Participants Characteristics

A total of 520 military personnel participated in the survey. The basic characteristics of the study sample are presented in Table 1. Most of the participants were males (486 individuals, representing 93.5% of the sample), as shown in Table 1. Female participants were significantly fewer, with only 34 individuals making up 6.5% of the total sample.

### 3.2. Exploratory Factor Analysis

The initial analysis starts with an exploratory factor analysis (EFA) to examine the factor structure of the Arabic-MBI-MP. A total of 22 items were included in the EFA to evaluate the underlying dimensions of burnout. The suitability of the data for factor analysis was confirmed by the Kaiser–Meyer–Olkin (KMO) measure of sampling adequacy (KMO = 0.924), which exceeded the minimum acceptable value of 0.50 (Hair et al., 2014). Bartlett’s test of sphericity was significant (χ^2^ = 11713.34, df = 231, *p* < 0.001), indicating adequate correlations among the items.

**Figure 1:**
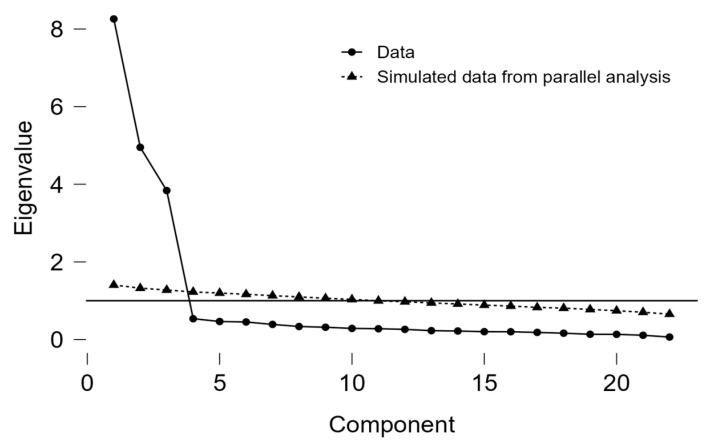
Scree plot confirming the three-factor structure of MBI among military personnel.

To determine the number of factors to extract, a scree plot was analyzed (Figure 1). The objective of the scree plot was to identify factors with eigenvalues greater than 1, where the intersection with the line perpendicular to the eigenvalue axis indicated the cut-off point. The parallel analysis, conducted with 1000 simulated random data sets, supported a three-factor solution, which corresponded to emotional exhaustion (EE), depersonalization (DP), and personal accomplishment (PA). The three factors explained 57.1%, 28.6%, and 14.3% of the total variance, respectively, confirming the dimensional structure of the scale.

Following the scree plot examination, Table 2 displays the factor loadings for the MBI items on each identified factor. All items demonstrated strong loadings on their respective factors, supporting the three-factor structure of the Arabic-MBI-MP.

### 3.3. Confirmatory Factor Analysis

Following the EFA, a confirmatory factor analysis (CFA) was subsequently conducted to assess the construct validity of the Arabic-MBI-MP based on the three-factor structure identified in the EFA. The CFA model fit indices indicate that the hypothesized model fits the data well with Comparative Fit Index (CFI) = 0.949 (values above 0.90 indicate good fit, with values above 0.95 considered excellent), Tucker–Lewis Index (TLI) = 0.943 (indicating a good fit), Root Mean Square Error of Approximation (RMSEA) = 0.0742 (95% CI: 0.068–0.0797); values below 0.08 are acceptable, with values below 0.06 indicating close fit) and a Standardized Root Mean Square Residual (SRMR) = 0.062 (indicating good correspondence between predicted and observed data).

Although the chi-square value was significant (χ^2^ = 796, df = 206, *p* < 0.001), this is expected in large samples and does not necessarily indicate a poor model fit. The high CFI and TLI values, as well as the acceptable SRMR and RMSEA, suggest a good fit of the three-factor model to the data, demonstrating strong construct validity of the Arabic-MBI-MP among military personnel.

**Figure 2:**
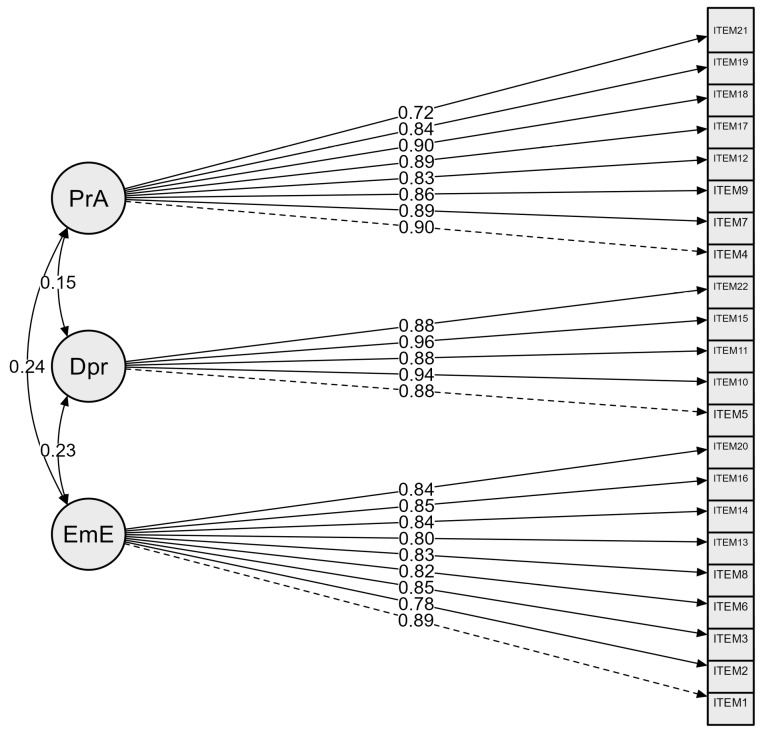
Confirmatory factor analysis of the MBI among military personnel. Comparative Fit Index (CFI) = 0.949; Tucker–Lewis Index (TLI) = 0.943; Root Mean Square Error of Approximation (RMSEA) = 0.0742, and Standardized Root Mean Square Residual (SRMR) = 0.062; Personnel Accomplishment (PrA), Depersonalization (Dpr), Emotional Exhaustion (EmE).

Figure 2 presents the CFA model, and Table 3 summarizes the model fit indices, showing that the standardized coefficients between factors and items are all above 0.60, ranging from 0.68 to 0.94.

To strengthen the evidence for the construct validity, we calculated the Average Variance Extracted (AVE) for each factor. AVE values of 0.5 or higher were considered highly satisfactory, while a value of 0.5 was considered acceptable. The AVE values for each factor were as follows: emotional exhaustion (EE) [AVE = 0.65], depersonalization (DP) [AVE = 0.68], and personal accomplishment (PA) [AVE = 0.70]. These AVE values support the construct validity of the Arabic-MBI-MP by demonstrating that each factor captures a substantial proportion of variance in its associated items.

### 3.4. Reliability Analysis

The reliability of the Arabic-MBI-MP was evaluated through the assessment of internal consistency using Cronbach’s alpha and McDonald’s omega, showing the following values: emotional exhaustion (EE): α = 0.844, ω = 0.844; depersonalization (DP): α = 0.919, ω = 0.910; and personal accomplishment (PA): α = 0.890, ω = 0.890. In addition, deleting any item from these constructs did not significantly affect the Cronbach’s alpha values, confirming strong internal consistency (Table 4).

## 4. Discussion

This study validated the Maslach Burnout Inventory (MBI-HSS) among Tunisian military personnel, Arabic-MBI-MP, by assessing the factor structure, construct validity, and reliability analysis.

The exploratory factor analysis showed a three-component structure: emotional exhaustion, depersonalization, and personal accomplishment; moreover, no elements were deleted from the measurement scale. The internal consistency indices and corrected item-total correlation were employed to evaluate the instrument’s reliability. The findings demonstrated that all three dimensions of the instrument were robust and represented the concepts well. The confirmatory factor analysis indicated a second-order structure with satisfactory fit indices. The construct validity of the measurement tool was confirmed.

The initial statistical analysis demonstrated that the measuring scale and the associated data were appropriate for conducting the EFA. The actual data from the 22 items demonstrated a robust correlation with the three-factor model established by the original MBI: emotional exhaustion, depersonalization, and accomplishment. Consequently, there has been no elimination of any item. These results aligned with the findings of previous studies that developed and validated the measure (Córdoba et al., 2011; Gil-Monte, 2005; Loera et al., 2014).

The internal consistency of the Maslach Burnout Inventory A-MBI-MP was evaluated using Cronbach’s alpha and McDonald’s ω, showing high reliability across all three subscales. The emotional exhaustion (EE) subscale revealed an alpha of 0.844, showing that the items assessing emotional fatigue are reliably associated and represent a cohesive construct. The depersonalization (DP) subscale showed an alpha of 0.919, signifying excellent internal consistency, while the personal accomplishment (PA) subscale recorded an alpha of 0.890, reflecting robust reliability. These findings correspond with the established standard for acceptable reliability, generally established at 0.70 or above for Cronbach’s alpha (Considine et al., 2005). The elevated scores recorded for the MBI subscales validate the instrument’s reliability in evaluating burnout among Tunisian military personnel. Further, these findings are consistent with prior research by Maslach and colleagues, who confirmed the internal consistency of the MBI in different occupational categories, indicating Cronbach’s alpha values of 0.90 for the emotional exhaustion subscale and 0.79 for the depersonalization subscale, therefore showing the MBI’s strong reliability across multiple contexts. The personal accomplishment subscale usually shows a little lower reliability in certain populations, with alpha values between 0.70 and 0.82 in studies of healthcare workers and educators (Maslach & Jackson, 1996).

The results also aligned with findings from other Arabic-speaking populations and diverse cultural situations. A study in Lebanon dentists indicated comparable Cronbach’s alpha values for emotional exhaustion (EE = 0.85), depersonalization (DP = 0.82), and personal accomplishment (PA = 0.66) (Bassam et al., 2023). The findings indicate that the MBI when translated and converted to Arabic, preserves its internal consistency and applicability in assessing burnout across various professions and cultural situations. The model fit statistics, including the CFI, TLI, RMSEA, and SRMR, were all indicative of a good fit; however, the chi-square test typically indicates model fit for sample sizes between 75 and 200 when the sample size surpasses 400, the test’s heightened sensitivity frequently results in the rejection of most models, hence diminishing its applicability in such cases (Stone, 2021). These results are in line with a review and meta-analysis of 45 exploratory and confirmatory factor-analytic by Worley et al. (Worley et al., 2008), which provide support for the concept that PA is a distinct component in the A-MBI-MP as well. The negative relationship between the PA and the two burnout subscales (emotional exhaustion and depersonalization) exhibits consistency. In addition, the affirmative correlation between FPB “feel” the patient’s blame and the two burnout subscales (emotional exhaustion and depersonalization) appears to align with the findings of González-Romá et al. (2006) designated as “identification”. The connections between occupational burnout and work engagement have also been addressed and emphasized by more researchers (Demerouti et al., 2010). Burnout has been described in various ways, going from an emotional response toward chronic workplace stress to a syndrome of emotional exhaustion, depersonalization, and reduced personal accomplishment (Leiter & Maslach, 2017; Maslach & Leiter, 2016). The variability of these definitions confuses whether burnout is a distinct construct or overlaps significantly with other psychological phenomena, such as depression, stress, or fatigue (Bianchi et al., 2015; Leiter & Maslach, 2016).

*How can a tool such as the MBI offer an evaluation of burnout with the construct’s ambiguity?*

The MBI is based on Maslach and Jackson’s (1981) definition, which characterizes burnout as a three-dimensional condition: emotional exhaustion, depersonalization, and diminished personal accomplishment (Maslach & Jackson, 1981). This model does not include all definitions of burnout found in the literature (Todaro-Franceschi, 2024), and some researchers argue that it may not adequately reflect the complexity of the burnout experience across various professions or cultural contexts (De Beer et al., 2024; Hassankhani et al., 2024; Kristensen et al., 2005). The focus on depersonalization in the MBI may not apply completely to every occupational situation, creating questions about its ability to be generalized (Taris et al., 2005).

*How far can the A-MBI-MP measure burnout?*

The MBI designers are partially accurate in asserting that weariness, cynicism, and inefficacy need to be addressed independently (Bianchi et al., 2023). Also, the three entities cannot be readily categorized under a generic or higher-order “burnout” factor; although the MBI is a comprehensive instrument, it may not encompass all aspects of stress and burnout pertinent to military professions. Future research may investigate military-specific burnout measures or integrate the MBI with additional evaluations to provide a more comprehensive understanding of mental health within this population.

The issues impacting the MBI may be expected, considering the origins of the burnout concept. Burnout originated in the mid-1970s as a mostly developed concept. The definition of burnout was not based on solid empirical research or solid conceptual frameworks, nor did it result from a comprehensive literature review on stress and health (Bianchi et al., 2024). Further, the definition seems to be inherently contradictory; burnout, as a syndrome (Bridgeman et al., 2018), is expected to arise from the interplay of its three components: exhaustion, cynicism, and inefficacy; however, this combination is considered unsuitable and explicitly not allowed. Investigators ultimately have three distinct constructions, none of which, as stated by the MBI developers, can be equated with burnout (Bianchi et al., 2024).

### 4.1. Practical Implications

The validation of the MBI in the military context provides practical implications for military healthcare personnel and leaders. According to the assessment’s reliability, it can be utilized to evaluate burnout levels in Tunisian military personnel, facilitating early detection and intervention. Prior research indicates that burnout correlates with diminished job performance, higher leave rates, and negative health consequences (Leiter & Maslach, 2005). Considering that the psychometric properties of the A-MBI-MP were carefully assessed and produced satisfactory results, military personnel can utilize the A-MBI-MP to determine burnout levels. Consequently, early therapies or interventions may be undertaken to avert significant health issues resulting from burnout among military personnel, and the current findings have specific relevance in the following domains (Clifford, 2014). The assessment of military burnout can be improved and given more precisely based on the good psychometric features identified in this. Additionally, the validation of the Arabic version of the Maslach Burnout Inventory (MBI) offers military leaders and mental health experts a consistent instrument to identify burnout early, thereby enabling timely interventions to avert psychological suffering and performance drop. It helps the creation of specifically targeted mental health initiatives addressing burnout-related issues experienced by military personnel speaking Arabic. Effective burnout management can help military forces improve operational readiness and guarantee that members remain physically and psychologically ready for duty. Furthermore, the validated MBI guides policy formulation on stress reduction and task management, thereby promoting better and more environmentally friendly military personnel. Moreover, it enables military leaders to improve their leadership approaches to create a conducive atmosphere, raise morale, and reduce personnel’s stress.

### 4.2. Strengths and Limitations

To the authors’ knowledge, this study is the first to examine the reliability and validity of the Arabic version of the MBI-HSS scale among Tunisian military personnel. It provides important insights into the validity of the MBI among Tunisian military personnel. However, there are certain limitations to the study when interpreting the findings.

First, the study was one-shot research, which limited our capacity to follow the progression of burnout levels over time. Hence, using a longitudinal design would provide a more profound comprehension of the development and variability of burnout in military contexts (Kim, 2021). Second, all participants were engaged in full-time jobs; consequently, the findings cannot be applied to individuals in part-time employment. Future studies could similarly examine the comparison of measurement invariance based on employment type (Xu, 2020).

Finally, the study on gender variations in burnout produced contradictory findings concerning the size and trajectory of this association. Additionally, the absence of clarity regarding gender differences in organizationally significant phenomena, such as work burnout, often leads to unfounded speculations that may result in a lack of knowledge for organizational decisions (Purvanova & Muros, 2010). Henceforth, future research could assess the invariance of the A-MBI-MP according to gender, even though the sample size of military females almost worldwide may be non-representative and problematic for the assessment of gender invariance. Furthermore, the concurrent or discriminant validity of the A-MBI-MP could be investigated in future studies to explore the complexity of military staff’s understanding of burnout.

## 5. Conclusions

The current study provides that the Maslach Burnout Inventory A-MBI-MP is a relevant and reliable instrument for evaluating burnout levels in Tunisian military personnel. The findings validate the cross-cultural relevance of the MBI, highlighting its efficacy in assessing the three factors of MBI. This validation adds to the increasing literature on burnout assessment in many contexts and highlights the necessity of managing burnout in the military setting. Moreover, ignoring burnout can have profound implications for individual well-being, unit cohesion, and mission effectiveness. Further research needs to investigate the applicability of the MBI across many military settings and cultural contexts to improve its generalizability.

## Figures and Tables

**Table 1:** Sociodemographic characteristics of participants.

		Frequency	Percentage %
Gender	Male	486	93.5
	Female	34	6.5
Age	20 to 52 years with an average of 27.7 ± 6.9 years		
Marital Status	Single	389	74.8
	Married	123	23.7
	Divorced	7	1.3
	Widower	1	0.2
Specialty	Commandos	214	41.2
	Pilot	203	39.0
	Diver	103	19.8
Total	520		100%

**Table 2:** Exploratory factor analysis of the Arabic-MBI-MP.

	Factor Loading for the MBI Items		
	Emotional Exhaustion (EE)	Depersonalization (DP)	Personal Accomplishment (PA)
Item 1	0.878		
Item 2	0.819		
Item 3	0.859		
Item 20	0.845		
Item 8	0.826		
Item 13	0.815		
Item 14	0.859		
Item 6	0.851		
Item 16	0.846		
Item 5		0.897	
Item 10		0.943	
Item 11		0.905	
Item 15		0.947	
Item 22		0.903	
Item 4			0.910
Item 7			0.889
Item 9			0.869
Item 12			0.860
Item 17			0.886
Item 18			0.907
Item 19			0.854
Item 21			0.952

**Table 3:** The fit indices for the model of Arabic-MBI-MP.

				IC90% RMSEA			
CFI	TLI	SRMR	Approximation	Lower	Upper	AIC	χ^2^
0.949	0.943	0.062	0.0742	0.0688	0.0797	31,229	796

Notes: χ^2^: chi-square, CFI: Comparative Fit Index, TLI: Tucker–Lewis Index, SRMR: Standardized Root Mean Square Residual, RMSEA: Root Mean Square Error of Approximation.

**Table 4:** Internal consistency of the Arabic-MBI-MP.

			If the Item Is Deleted	
	Average	Standard Deviation	Cronbach’s α	McDonald’s ω
Item 1	3.27	1.80	0.913	0.913
Item 2	3.51	1.83	0.916	0.916
Item 3	3.43	1.80	0.915	0.915
Item 6	3.56	1.74	0.915	0.915
Item 8	3.46	1.87	0.914	0.914
Item 13	3.43	1.83	0.915	0.915
Item 14	3.50	1.75	0.915	0.915
Item 16	3.40	1.78	0.914	0.914
Item 20	3.45	1.75	0.914	0.914
Item 5	3.27	1.16	0.919	0.918
Item 10	3.40	1.20	0.919	0.919
Item 11	3.44	1.13	0.920	0.919
Item 15	3.33	1.42	0.919	0.918
Item 22	3.46	1.17	0.920	0.919
Item 4	3.30	1.51	0.916	0.916
Item 7	3.49	1.45	0.916	0.915
Item 9	3.45	1.42	0.916	0.916
Item 12	3.16	1.58	0.917	0.917
Item 17	3.46	1.49	0.916	0.915
Item 18	3.46	1.49	0.917	0.916
Item 19	3.47	1.52	0.917	0.916
Item 21	3.38	1.62	0.919	0.918

## Data Availability

The original contributions presented in the study are included in the article. Further inquiries can be directed at the corresponding author.
